# Co-administration of human adipose-derived stem cells and low-level laser to alleviate neuropathic pain after experimental spinal cord injury

**DOI:** 10.1186/s13287-019-1269-y

**Published:** 2019-06-24

**Authors:** Arash Sarveazad, Atousa Janzadeh, Gholamreza Taheripak, Sima Dameni, Mahmoud Yousefifard, Farinaz Nasirinezhad

**Affiliations:** 1 Colorectal Research Center, Iran University of Medical Sciences, Tehran, Iran; 2 Radiation Biology Research Center, Iran University of Medical Sciences, Tehran, Iran; 3 Department of Biochemistry, Faculty of Medicine, Iran University of Medical Sciences, Tehran, Iran; 4 Physiology Research Center, Faculty of Medicine, Iran University of Medical Sciences, Tehran, Iran; 5 Physiology Research Center and Department of Physiology, Faculty of Medicine, Iran University of Medical Sciences, Tehran, Iran; 6 Department of Physiology, School of Medicine, Iran University of Medical Sciences, Hemmat Highway, Tehran, Iran

**Keywords:** GSK3 beta, Neurodegeneration, Neuropathic pain, Hyperalgesia, Allodynia, Spinal cord injury

## Abstract

**Background:**

Evidence has suggested that human adipose-derived stem cells (hADSCs) and low-level laser has neuroprotective effects on spinal cord injury (SCI). Therefore, the combined effect of the hADSCs and laser on neuregeneration and neuropathic pain after SCI was investigated.

**Methods:**

Forty-eight adult male Wistar rats with 200–250 g weight were used. Thirty minutes after compression, injury with laser was irritated, and 1 week following SCI, about 1 × 10^6^ cells were transplanted into the spinal cord. Motor function and neuropathic pain were assessed weekly. Molecular and histological studies were done at the end of the fourth week.

**Results:**

The combined application of hADSCs and laser has significantly improved motor function recovery (*p* = 0.0001), hyperalgesia (*p* < 0.05), and allodynia (p < 0.05). GDNF mRNA expression was significantly increased in hADSCs and laser+hADSC-treated animals (*p* < 0.001). Finally, co-administration of hADSCs and laser has enhanced the number of axons around cavity more than other treatments (*p* < 0.001).

**Conclusions:**

The results showed that the combination of laser and ADSCs could significantly improve the motor function and alleviate SCI-induced allodynia and hyperalgesia. Therefore, using a combination of laser and hADSCs in future experimental and translational clinical studies is suggested.

## Background

Spinal cord injury (SCI) is one of the most common and complicated clinical problems around the world and brings about many disabling outcomes such as motor dysfunction, neuropathic pain, and social problems [1]. Pain due to SCI undoubtedly affects the individual’s quality of life, and the priority of many SCI patients is relieving the pain instead of developing the ability to walk [2, 3]. Chronic pain following SCI is very common, and 75–80% of SCI patients experience this pain [4–6]. Based on the classification of the International Association for the Study of Pain (IASP), SCI pains are divided into two groups: nociceptive (skeletal, muscular, and visceral pains) and neuropathic (pains due to injury or dysfunction of the neural system) [2]. Treatment of neuropathic pain is different from nociceptive ones. Various classes of drugs with analgesic characteristic are applied for treating neuropathic pain [7]. In addition to drug therapy, non-drug treatments for neuropathic pain such as surgery, acupuncture, transcutaneous electrical nerve stimulation, psychotherapy, and physiotherapy exist [2, 8–13]. Low-level laser therapy is one of the physiotherapy measures that can be taken for controlling neuropathic pain, and it has recently received much attention from researchers due to its low cost, being non-invasive and not having side effects [14, 15]. Considering the anti-inflammatory property of laser [15, 16], during inflammation, laser results in control of neuropathic pain via balancing chemical mediators, dilating arteries, increasing cortisol synthesis [15, 17, 18], and increasing the endorphin synthesis [19, 20].

Cell therapy is another non-drug measure that is currently the center of attention for treating neural diseases [21–23] including neuropathic pain [24, 25]. Clinical use of mesenchymal stem cells (MSCs) with the aim of treating various diseases [22, 26–28] is increasing day by day since its safety and efficacy have been proven [29, 30]. Adipose tissue has received more attention from researchers for extracting stem cells compared to older sources of MSCs because it is available more easily, is safer, and has richer stem cell content [31]. Human adipose-derived stem cells (hADSCs) can be a proper choice for treating neuropathic pain since they secrete cytokines [32, 33] and improve nerve healing [34]. Considering the anti-inflammatory and nerve regenerating properties of laser and hADSCs, the combination of these two can be considered as an effective non-drug treatment modality in controlling and treating of SCI induced neuropathic pain.

It seems that single-therapy do not have a sufficient effect on the SCI [35]. In recent years, several preclinical studies used combination of various treatments for SCI [36–38]. Based on our knowledge, no study has investigated the effect of combination of laser and hADSCs on the recovery after SCI. Therefore, the aim of the present study was evaluating the effect of simultaneous prescription of laser and hADSCs on neuropathic pain after induction of SCI model in rats.

## Methods

### Studied animals

In this study, adult male Wistar rats (*n* = 48) with 200–250 g weight, which were obtained from the experimental studies center of Iran University of Medical Sciences, were used. The animals were randomly divided into six equal groups (*n* = 8) (Table 1). All the stages of this research project were carried out in Iran University of Medical Sciences (Tehran, Iran) after receiving the approval of the ethics committee (IR.IUMS.REC1395.95-04-30-27315). Animals were kept in the standard condition of animal house, had access to sufficient water and food, and were in + 21 °C and 12-h dark/light cycles before and during the study.

**Table 1:** The study groups

Study groups	Explanation
Control	Without intervention
SCI	SCI+ without intervention
Vehicle	SCI+ 10 μl PBS (in situ injection)
Laser	SCI+ Laser
hADSCs	SCI+ 10^6^ hADSCs suspended in 10 μl PBS (in situ injection)
Laser+hADSCs	SCI+ laser+10^6^ hADSCs suspended in 10 μl PBS (in situ injection)

*SCI* spinal cord injury, *hADSCs* human adipose-derived stem cells

### Isolation of hADSCs

Adipose tissue was extracted from the superficial fat layer of the abdomen during liposuction of individuals 25 to 35 years old (after obtaining consent) who had visited the general surgery room of the Rasoule Akram Hospital (Tehran, Iran) for undergoing liposuction surgery. According to their medical profile, these individuals had no history of HBS and HIV Ag. Sampling was done in sterile conditions and then transferred to the laboratory in a sterile dish that contained DMEM/Ham s F-12, FBS 10%, and penicillin/streptomycin (P/S) 5%. Isolation of stem cells was done based on the protocol described in detail by Dubois et al. [34]. For this purpose, the fat sample was warmed in a water bath up to 37 °C before extraction stages. After that, all the stages of extraction were performed under a sterile hood with sterile material and equipment. Two hundred milligrams of adipose tissue was placed in a tube containing penicillin/streptomycin (P/S) 1% (prepared with warm PBS) until blood vessels and connective tissue were isolated, and finally, the tissue became clear (usually two times washing). The sample was then transferred to a tube containing collagenase 0.1% and BSA 1% (prepared with warm PBS), for tissue digestion. The tube containing the sample was placed in a water bath for 30 min until the tissue was completely digested and the solution became clear. After tissue digestion, the tube containing the sample was centrifuged for 5 min with a speed of 1200 rpm in room temperature. After discarding the supernatant, the pellet formed was re-suspended using BSA 1% and centrifuged again. To eliminate red blood cells (RBCs), the pellet formed was re-suspended using RBC lysis buffer and was again centrifuged after pipetting. Finally, after washing with PBS, centrifuging, and discarding the supernatant, the formed pellet was re-suspended using DMEM/Ham s F-12 culture medium that contained FBS 10% and penicillin/streptomycin (P/S) 1% and was then transferred to a flask. Flasks were kept in an incubator (37 °C temperature, 5% CO2, 98% humidity) until the third passage.

### Identifying hADSCs

Identifying hADSCs was done in the third passage using flow cytometry. CD73, CD90, and CD105 were considered as positive markers, and CD34 and CD45 as negative markers. After trypsination, hADSCs were centrifuged for 5 min in 3000×*g*, and then for retrieving surface markers, 1 × 10^6^ hADSCs were incubated in DMEM-f12, 10% FBS, and 1% P/S at 37 °C in 5% CO2 for 4 h. In the next phase, cells were incubated with the conjugated primary antibody for 30 min. Then, after centrifugation, the cells were washed with PBS three times and divided into aliquot parts with 500 μl volume. Flow cytometry was performed using Cyflow Space flow cytometer (Sysmex-Partec). Antibodies used for flow cytometry were CD29-PE, CD73-PE, CD34-PE, CD105-FITC, and CD45-FITC as direct antibodies and IgG1-PE and IgG1-FITC as isotype control (BD Biosciences).

### Labeling hADSCs

For detecting hADSCs 4 weeks after implanting, cells were labeled via Dil. In summary: 1 × 10^6^ hADSCs were suspended in 1 ml DMEM-F12 containing 5 μl Dil (Invitrogen, C-7000, USA) solution (50 μg Dil in 50 μl DMSO) and incubated for 15 min in 37 °C, CO2 5%, humidity 98%, and then for 10 min in 8 °C. After centrifugation (5 min at 1200 RPM) and discarding the supernatant, the cells were re-suspended in PBS for injection.

### Surgery and confirmation of SCI model

After analgesia of the animals via intraperitoneal (IP) injection of ketamine (80 mg/kg) and xylasine (10 mg/kg), they were put in prone position, and after shaving the skin in the region of lumbar vertebra and prepping with betadine, a section was made in the middle line using a bistoury blade. After pushing subcutaneous fat and muscles aside and exposing lamina of the vertebra, laminectomy was performed at the level of T13-L1 in the spinal cord without injuring dura mater. Aneurysm clips made by FST Company that provide a force equal to 20 g/cm2 were used for 90 s to induce spinal cord injury via compression method. The muscles and skin were sutured with 0.3 suture thread. Post-surgery care included prescription of ringer solution for prevention of dehydration (3 ml, IP, after surgery), prescription of penicillin G for 4 days after surgery (8 mg/100 g, IP), and bladder massage twice a day was done for all the animals. Animals that had a Basso, Beattie, and Bresnahan (BBB) score higher than 3 3 days after SCI induction were excluded from the study. To confirm the injury model, an animal was deeply anesthetized via IP injection of ketamine and xylosine 1 week after induction of SCI, and after cardiac perfusion with paraformaldehyde 4%, a section of the spinal cord with 1.5 cm length, including the site of injury, was removed and placed in sucrose 30% overnight and molded in cryo protection medium (OCT). Using cryostat device, 10-μm-thick cross-sections were prepared for the site of injury.

### The method of 660 nm low-level laser therapy

In the present study, diode CW laser with 660-nm wavelength and 100-mW power that was received as a gift from the Heltschl Company (model ME-TL10000-SK) was used. The laser was fixed on a metal rod to maintain the same radiant distance throughout the study. Thirty-minutes after removing the aneurysm clip from the site of injury, which was marked before, 5 mm higher and 5 mm lower as well as the mirror of these points on the left and right of the site of injury were radiated with 660-nm low-level laser with 5 mm distances (9 points). On each point, the laser was radiated for 5 s (overall 45 s). Laser radiation with this method continued for 2 weeks.

### hADSCs implantation

One week after injury, the animals were anesthetized with the same method mentioned in the SCI section, and the spinal cord was opened in the T13-L1 region via a glass microelectrode that was attached to a Hamilton syringe; 10^6^ cells in a 10 μl volume were implanted in the dorsal horn of the spinal cord. The implant was done using a stereotaxy device. The injection was done in two places. In each injection, 5 μl of cell volume was implanted into the spinal cord. The sites of these injections were 0.5 mm higher and lower than the site of injury in the closest place to the central vein of the spinal cord with 1 mm depth. Then, the muscles and skin were sutured and the animals were put back in their cages.

### Behavioral assessment

Motor evaluation: motor function was assessed during 4 min using the open-field walking test. Each animal was allowed to freely move into a region 90 cm in diameter with a wall height of 24 cm for 4 min. The movement of the animal during the 4 min was recorded using a digital camera. Two independent observers watched the films and gave the animals scores from 0 (complete paralysis) to 21 (normal walking) based on the Basso Beattie and Bresnahan scale (BBB scale). The mean scores of the two observers were recorded as the score for each animal. One week after the injury, the BBB score of the animals was recorded. Only animals which had a BBB score less than 3 would be included in the study. BBB score was recorded for all the animals for 4 weeks [39].

### Pain assessment

#### Mechanical allodynia measurement test

Mechanical allodynia was measured by Von Frey filaments. The animal was placed on a metal mesh plate and inside a Plexiglas container with 40 × 40 cm dimensions about 60 cm above the ground level. When it got used to the environment, various Von Frey filaments (numbers 4.31, 4.56, 4.74, 4.93, and 5.18) were used to evaluate mechanical allodynia. The filaments were applied according to their thickness, and the thinnest was used first. They were vertically connected to the animal’s paw sole (up and down method). Filaments were applied with at least 20-s time intervals for each number and on both hind paws, then the mechanical pain threshold was calculated using Dixon software.

### Cold allodynia measurement test

Cold allodynia was measured by acetone test. In this method, the animal was placed on a wired network and a drop of acetone was splashed on the rat’s left hind paw using an insulin syringe. This test was done five times with 2-min intervals. Licking, shaking, or pulling the leg (2–5 s after application) were considered as positive response and were finally shown as a percentage.

### Mechanical hyperalgesia measurement test

Mechanical hyperalgesia was measured via Basile Analgesy Meter (Ugo Basile, Varese, Italy). Mechanical stimulation was done with a 48-g weight connected to a lever. The increasing pressure produced was determined using a ruler connected to the device. When the animal tried to respond to the applied pressure by pulling its hind paw, the pressure would stop and the amount of pressure that led the animal to respond was recorded. This test was carried out twice on each leg with at least 5-min time intervals, and the mean of the obtained results was recorded.

### Heat hyperalgesia measurement test

Heat hyperalgesia, was measured via plantar test (Ugo Basile, Varese, Italy). In this test, animals were placed in a clear container with a glass floor. After 15 min of accommodation, infrared glimmer was radiated to the animal’s paw. Responding with paw withdrawal would automatically stop heat radiation from the heat generated in the device. A 25-s cutoff was considered for preventing injury to the animal’s paw. This test was done three times in the injured foot with at least 5-min intervals, and the mean of the obtained measures was recorded as the response.

### Tissue evaluation

Four weeks after cell transplantation and laser therapy, the animals were deeply anesthetized via ketamine/xylosine. After cardiac perfusion with paraformaldehyde 4%, the part of the spinal cord that included the site of injury was removed (1.5 cm in length). After postfix (overnight paraformaldehyde 4%), the sample was transferred to sucrose 10%, 20%, and 30% solutions one overnight each, and then finally, after molding, serial 13-μm-thick cross-sections were prepared from the samples using a Cryo section device (histoline) for tissue studies. For tissue evaluations, five slides from each animal and five sections of the slide were selected.

### Cavity size and myelination assay

For cavity size and myelin area assay, Luxol Fast Blue (LFB) staining (stained in 0.1% solvent blue, Sigma, 38, S3382, USA) was used. In LFB staining, myelin including phospholipids look blue to green and neurons look pink to violet. Cavity size for each slide was recorded by ImageJ software, and eventually, the mean cavity size for each group was measured. According to our previous studies [40], the cavity size was presented as percent of the total area of the section using the following formula:

$$ \mathrm{Percentage}\kern0.5em \mathrm{of}\kern0.5em \mathrm{cavity}\kern0.5em \mathrm{size}=\frac{\mathrm{Cavity}\ \mathrm{size}\kern0.5em \left(\mathrm{Micrometer}\right)}{\mathrm{Total}\ \mathrm{area}\ \mathrm{of}\ \mathrm{the}\ \mathrm{section}\;\left(\mathrm{Micrometer}\right)}\times 100 $$

### Hematoxylin and eosinophil (H&E) assay

H&E staining was performed for assessment of fibroblast invasion into cavity of the injury site. After staining, digital images were captured (Olympus, magnification × 40) and invasion of fibroblast was quantified manually via ImageJ software.

### Number of axons around the cavity

For an assessment of the axon position around the cavity, Bielschowsky staining was performed. In this staining method, silver nitrate (Sigma, 7761-88-8, USA) was used to identify axons. Therefore, axons are seen in black. Slides were observed and captured by a light microscope equipped with a camera (Olympus, magnification × 20), and axon number and position were quantified manually by ImageJ software.

### Western blot analysis

Four animals were selected from each group. After carefully removing the dura from an injured section of spinal cord, the spinal cord was homogenized using a buffer (Universal DNA/RNA/Protein Purification kit, EURx, Poland), then protein concentrations were determined by the nanodropper (Thermo Science, USA). Equal amounts of protein per wells were separated by 12% SDS-polyacrylamide gel (SDS-PAGE-Bio-Rad, USA) for 1 h at a constant voltage (120 V). Then, the bands were transferred to polyvinylidene-difluoride (PVDF) membranes. The membranes were blocked in PBS containing 0.05% Tween-20 (PBST) and 5% non-fat dry milk for 4 h at 37 °C. Then, the membrane was washed three times with PBST and then incubated with the rabbit polyclonal to aquaporin 4 (1/1000, abcam, ab46182, UK), gad65 (1/500, biorbyt, orb10682, UK), GSK3 β (1/300, biorbyt, orb89070, UK), and β-actin (1/1000, abcam, ab8227, UK) overnight at 4 °C in blocking buffer. After being washed with TBST (3 × 10 min), the membranes were incubated with goat anti-rabbit horseradish peroxidase-conjugated IgG (1/2000; abcam, ab6721, UK) for 2 h at room temperature. The protein bands were visualized by enhanced chemiluminescence (ECL) and exposed against X-ray film in the darkroom. Densitometry analysis for proteins was done using the Alpha EaseFC software.

### Immunohistochemistry

Prepared sections were washed three times with PBS, Triton X-100/10%, and subsequently blocked with BSA 1% and normal goat serum for 1 h at room temperature. The primary rabbit polyclonal antibodies GSK3 β (1/200, biorbyt, orb89070, UK), GABAB receptor1 (1/1000, abcam55051, UK), and anti-GABAB receptor 2 (1/1000, abcam52248, UK) were diluted in blocking solution and incubated overnight. Secondary antibody was goat anti-rabbit FITC (1/1000, abcam, ab6717, UK) diluted in PBS/0.3% BSA incubated for 1 h, respectively. Images were captured from each section (red due to DiI and green due to secondary antibody) under fluorescent microscope (magnification × 10). According to our previous studies [40, 41], the reactive area (fluorescent) was identified and the area was measured using ImageJ software based on the following formula:

$$ \mathrm{Percentage}\kern0.5em \mathrm{of}\kern0.5em \mathrm{immunoreactive}\kern0.5em \mathrm{area}=\frac{\mathrm{Immunoreactive}\kern0.5em \mathrm{area}}{\mathrm{Total}\ \mathrm{area}\ \mathrm{of}\ \mathrm{the}\ \mathrm{section}} \times 100 $$

### Quantitative real-time PCR

Total RNA was extracted from injured spinal cord tissues using Universal DNA/ RNA/Protein Purification kit (EURx, Poland), then RNA concentrations were determined using the nanodropper; cDNA was synthesized from isolated RNA via cDNA synthesis kit (EURx,E0801-02, Poland). Amplification and real-time detection were performed on an ABI model Stepone (Thermo fisher scientific, USA) using SG qPCR Master mix (EURx, E0402-01, Poland) and QuantiTect primers (Cinna Gen, Iran). The improved four-step reaction was used: 95 °C 10 min; 95 °C 10s, 60 °C 30 s, 72 °C 30 s, and 85 °C 15 s, for 40 cycles; the melting curve analysis was done with the temperature ranging from 60 to 95 °C, gradually increasing at a speed of 0.5 °C every 10 s. Relative quantitative analysis of the final results was normalized to beta-actin (Table 2).

**Table 2:** Sequence of primer pairs for real-time PCR

Primer name	Sequence 5′-3′
IL-6 (forward)	GACTGATGTTGTTGACAGCC
IL-6 (reverse)	CTGACAGTGCATCATCGCTG
BDNF (forward)	GGCTGACACTTTGAGCACG
BDNF (reverse)	GCTGTGACCCACTCGCTAA
GDNF (forward)	TATGGGATGTCGTGGCTGTC
GDNF (reverse)	CGCCGCTTGTTTATCTGGTG
Beta-actin (forward)	GGCAAGGTCATCCCAGAGC
Beta-actin (reverse)	CATCATACTTGGCAGGTTTCTCC

*IL-6* interlukin6, BDNF brain-derived neurotrophic factor, *GDNF* glial cell line-derived neurotrophic factor

### Statistical analysis

All the data were analyzed by prism 6 and SPSS version 21.0 (SCR:002865) then were presented as the means ± SEM. The differences of BBB and pain, behavioral tests between the studied groups, were evaluated by two-way ANOVA. Assessment of differences in histological and molecular evaluation was done by one-way ANOVA. For all analysis, a Bonferroni post hoc test was used to evaluate between group differences. Probability values (*p*) < 0.05 were measured to represent significant difference.

## Results

### Mortality

A total of 53 animals was included. During the induction of SCI, five animals died in the hADSCs (two rats) and vehicle (three rats) groups. Accordingly, data from 48 animals were included in the final analysis.

### Cell culture and flow cytometry assay

**Fig. 1:**
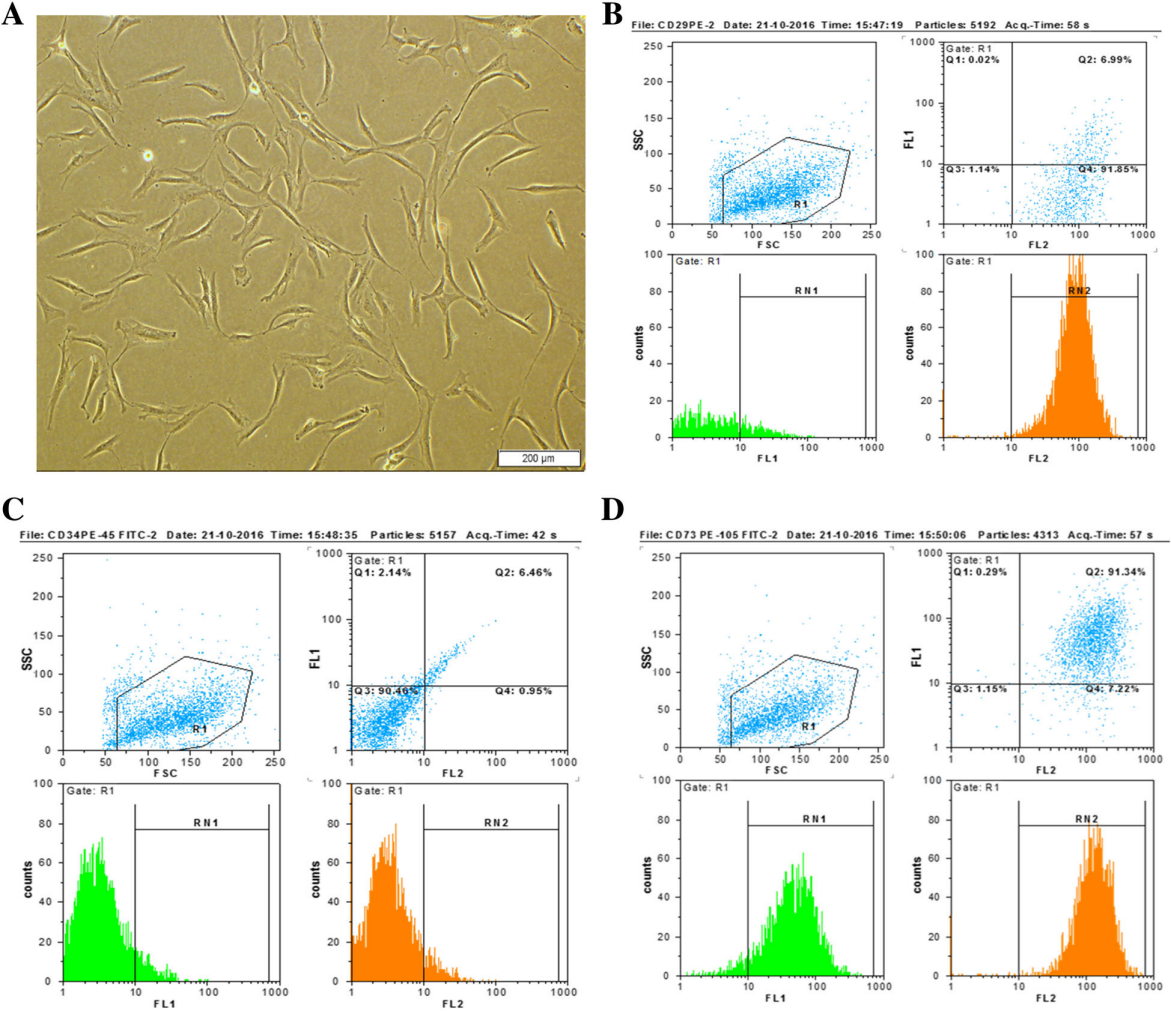
Cell culture and flow cytometry assay. **a** Flattened and spindle shape of hADSCs in the third passage, inverted microscopy, × 100. **b**–**d** Wide expression of CD29, CD73, and CD105 (positive) and very low expression of CD34 and CD45 (negative)

At the end of the third passage, hands were sticking to the floor of the flask and had a flattened and spindle shape (general morphology of mesenchymal stem cells) (Fig. 1a). Flow cytometry assay showed that hADSCs widely express CD29 (92.85%), CD73 (95.25%), and CD105 (96.02%) and very low express CD34 (7.02%) and CD45 (8.95) in the third passage (Fig. 1b–d).

### Behavioral assessment

#### Mechanical allodynia

**Fig. 2:**
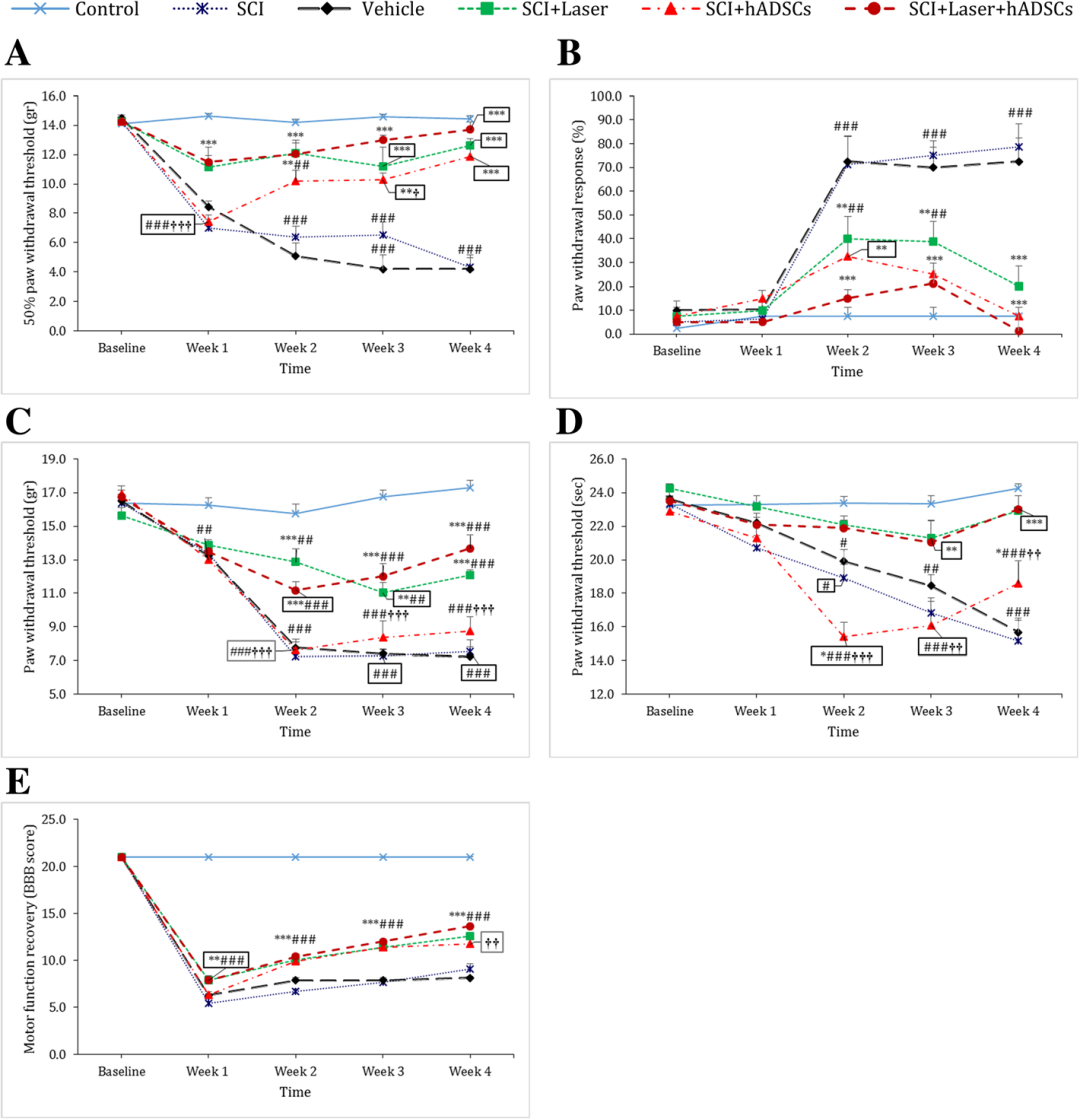
Effect of intraspinal injection of human adipose-derived stem cells (hADSCs) and low-level laser therapy (laser) alone and combination therapy on mechanical allodynia (**a**), cold allodynia (**b**), mechanical hyperalgesia (**c**), heat hyperalgesia (**d**) and (**e**) motor function recovery after spinal cord injury (SCI). Data are expressed as mean ± SEM (*n* = 8 in each group). *p < 0.05, **p < 0.01, ***p < 0.001 versus SCI groups. ^#^*p* < 0.05, ^##^*p* < 0.01, ^###^*p* < 0.001 versus control group. ^$^*p* < 0.05, vs laser group

The result showed that SCI caused a reduction in paw withdrawal threshold (df = 20, 168; F = 10.78; *p* < 0.0001). Treatments with laser (*p* < 0.0001) and hASDCs (*p* < 0.001 from week 2) and laser+hADSCs (*p* < 0.0001) improved paw withdrawal threshold compare to SCI. There is no significant difference between the laser+hADSC and control groups from week 2 to week 4 (*p* > 0.99) (Fig. 2a). The paw withdrawal threshold was higher in the laser+hADSC group than the hADSC group in the first (*p* < 0.0001) and third (*p* = 0.03) weeks.

### Cold allodynia

Two weeks after SCI induction, percentage of paw withdrawal responses to cold stimulation by acetone significantly increased in SCI and vehicle groups (df = 20, 168; F = 9.12; *p* < 0.0001). Treatments by laser (*p* < 0.001), hADSCs (*p* < 0.001), laser+hADSCs (*p* < 0.0001) decreased cold allodynia compared to SCI. The paw withdrawal response was reached to control group in all treated animals fourth week after SCI (*p* > 0.99) (Fig. 2b).

### Mechanical hyperalgesia

Paw withdrawal threshold to painful mechanical stimulation significantly improved after laser and laser+hADSC therapies compared to SCI group (df = 20, 168; F = 11.84; *p* < 0.0001). Treatment with hADSCs was not able to improve pain threshold (*p* > 0.99). Pain threshold in all the treated animals was lower than that in the control group (*p* < 0.01) (Fig. 2c). The paw withdrawal threshold was higher in the laser+hADSC than in the hADSC group after 2 weeks of SCI (*p* < 0.0001).

### Thermal hyperalgesia

Results showed that SCI led to a significant decrease in paw withdrawal threshold due to heat stimulation during 4 weeks (df = 20, 168; F = 6.75; *p* < 0.0001). hADSC transplantation improved thermal hyperalgesia in the second (*p* = 0.03) and fourth (*p* = 0.04) weeks compared to the SCI group. Treatment with laser (*p* = 0.001 for the third week and *p* < 0.0001 for the fourth week) and laser+ hADSCs (*p* = 0.004 for the third week and *p* < 0.0001 for the fourth week) improved paw withdrawal threshold to noxious heat stimulation compered to SCI animals. Pain threshold of laser-treated and laser+hADSC-treated animals had not significant difference compered to control group (*p* > 0.99) (Fig. 2d). Laser+hADSC-treated animals exhibited higher pain threshold compared to hADSCs group in the second (*p* < 0.0001), third (*p* = 0.0003), and fourth weeks (*p* = 0.002) after SCI.

### Motor function recovery

After SCI, motor function was significantly reduced in all groups compared to the control rats (df = 20, 168; F = 92.31; *p* < 0.0001). During the 4-week follow-up, some degree of motor function recovery was observed in the laser (*p* < 0.001), hADSC (*p* < 0.001), and laser+hADSC (*p* < 0.001) groups, but it did not reach the level of the control group (*p* < 0.0001). In the first week following SCI, a significant improvement was observed in the laser and laser+hADSC groups compared to SCI (*p* < 0.001). In the second to fourth weeks, BBB scores of all treated animals were higher than those in the SCI group (*p* < 0.0001) (Fig. 2e). The effect of laser+hADSCs on motor function recovery was higher than hADSCs in the fourth week (*p* = 0.0001).

### Glycogen synthase kinase-3β (GSK3β) in the spinal cord

**Fig. 3:**
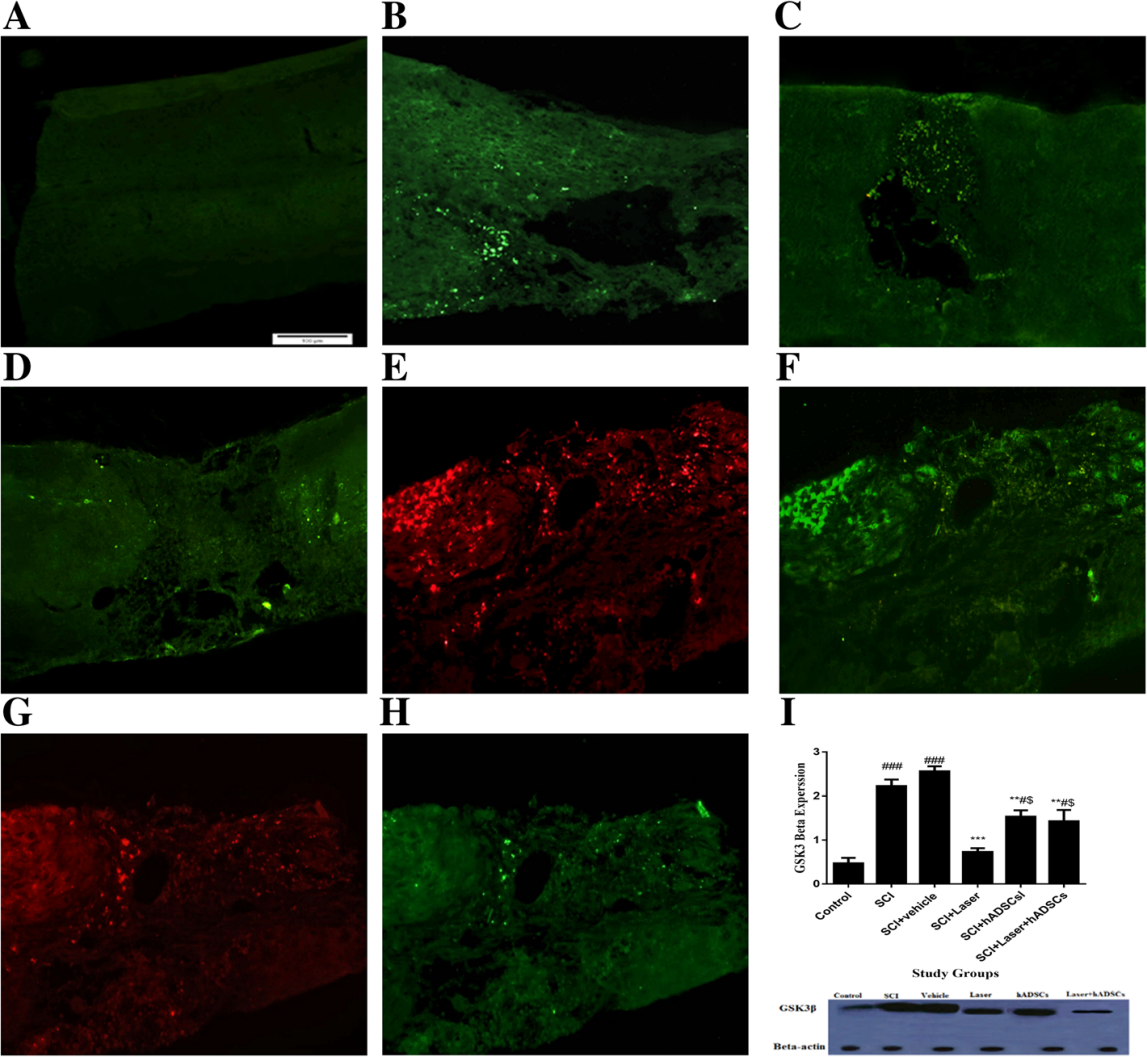
Localization (**a**–**h**, immunohistochemistry) and quantification (**i** and **j**, Western blotting) of glycogen synthase kinase-3β (GSK 3β) expression in experimental groups 4 weeks after spinal cord injury (SCI), the spinal cord longitudinal section (× 20). Control (**a**); SCI (**b**); vehicle (**c**); laser (**d**); human adipose-derived stem cells (hADSCs; cells labeled by DiI, red) (**e**); hADSCs (anti-anti GSK 3β conjugated whit FITC, green) (**f**); laser+hADSCs (red) (**g**); Laser+hADSCs (green) (**h**); Western blot of GSK3β expression (**i**). Data are expressed as mean ± SEM. *p < 0.05, **p < 0.01, ***p < 0.001 versus SCI groups. ^#^*p* < 0.05, ^##^*p* < 0.01, ^#^*p* < 0.001 versus control group. ^††^*p* < 0.01 vs combination group

GSK3β expressions significantly increased after SCI induction compared to the control animals (df = 5, 12; F = 28, 39; *n* = 3; *p* < 0.001). Laser irradiation (*p* = 0.0002), transplantation of hADSCs (*p* = 0.01), and laser+hADSCs (*p* = 0.004) reduced the expression of GSK3β (Fig. 3).

### Interleukin-6 (IL-6) gene expression

**Fig. 4:**
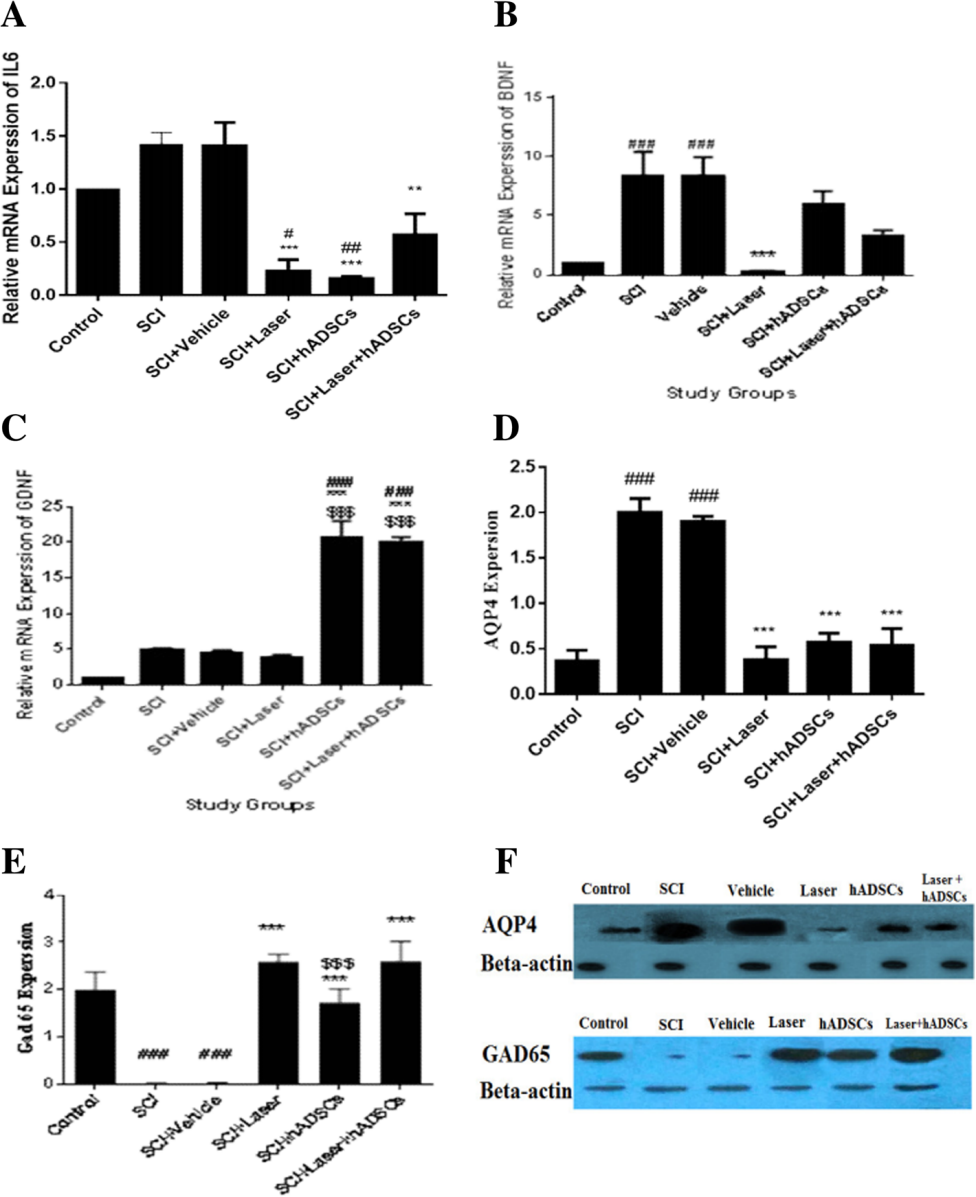
Interleukin6 (IL-6), brain-derived neurotrophic factor (BDNF), glial cell line-derived neurotrophic factor (GDNF) mRNA expression (**a**–**c**, real-time PCR) and aquaporin 4 (AQP4) and glutamic acid decarboxylase 65 (gad65) protein expression (**d**–**f**, Western blotting) 4 weeks after spinal cord injury (SCI) in the experimental groups. IL-6 (**a**); BDNF (**b**); GDNF (**c**); AQP4 (**d**, **f**); gad65 (**e**, **f**); western bolt band of AQP4 and gad65 (**f**), Data are expressed as the mean ± SEM. ^≠^*p* < 0.05 and ^≠≠^*p* < 0.01, ^≠≠≠^*p* < 0.001, vs control group, **p < 0.01, ***p < 0.001, vs. SCI group and vehicle, ^$$^*p* < 0.01, ^$$$^*p* < 0.001vs laser group

Analysis of IL-6 gene expression showed, 4 weeks after spinal cord injury, there was no difference in IL-6 gene expression compared to control animals (df = 5, 12; F = 16.77; *p* > 0.05, *n* = 3). Treatment with laser and hADSCs decreased its expression compared to the SCI and control (*p* < 0.001). Laser+hADSCs decreased IL-6 gene expression compared to SCI (*p* < 0.01) while, it remained at the control level (Fig. 4a).

### Brain-derived neurotrophic factor (BDNF) mRNA expression

Real-time PCR analysis of the spinal cord showed that mRNA level of BDNF decreased after induction of injury (df = 5, 12; F = 9.34; *p* < 0.001). Administration of laser decreased gene expression level of BDNF compared to SCI (*p* < 0.001). Transplantation of hADSCs did not change BDNF transcription compared to SCI and control. In hADSC-treated animals, BDNF mRNA expression was significantly higher than in the laser group (*p* = 0.047). However, the BDNF mRNA expression of the laser group had no significant difference with laser+hADSCs group (*p* = 0.50) (Fig. 4b).

### Glial cell line-derived neurotrophic factor (GDNF) mRNA expression

Real-time PCR analysis of spinal cord showed that mRNA level of GDNF in SCI (*p* = 0.27) and vehicle (*p* = 0.48) groups were not different with that of control group. Administration of laser did not cause changes in the expression level (*p* > 0.99). However, transplantation of hADSCs significantly increased GDNF transcription. In hADSC- (*p* < 0.001) and laser+hADSC- (*p* < 0.001) treated animals, GDNF mRNA expression was significantly higher than in other groups (df = 5, 12; F = 69.36; *p* < 0.001) (Fig. 4c).

### Aquaporin 4 (AQP4) expression

Induction of SCI increased AQP4 expression compared to control (df = 5, 15; F = 77.87; *n* = 3; *p* < 0.001). Treatments with laser, hADSCs, and laser+hADSCs significantly decreased AQP4 expression after SCI (*p* < 0.001) (Fig. 4d, f).

### Glutamic acid decarboxylase 65 (GAD65) expression

Induction of SCI resulted in significant reduction of GAD65 expression compared to the control after 4 weeks follow-up (df = 5, 12; F = 52.57; *p* < 0.001). Treatment with laser, hADSCs, and laser+hADSCs significantly (*p* < 0.001) increased GAD65 expression after SCI (*p* < 0.001). There is no significant difference between laser and laser+hADSC groups in GAD65 expression level (*p* > 0.99) (Fig. 4e, f).

### Gamma-aminobutyric acid (GABA) B receptors 1 and 2

**Fig. 5:**
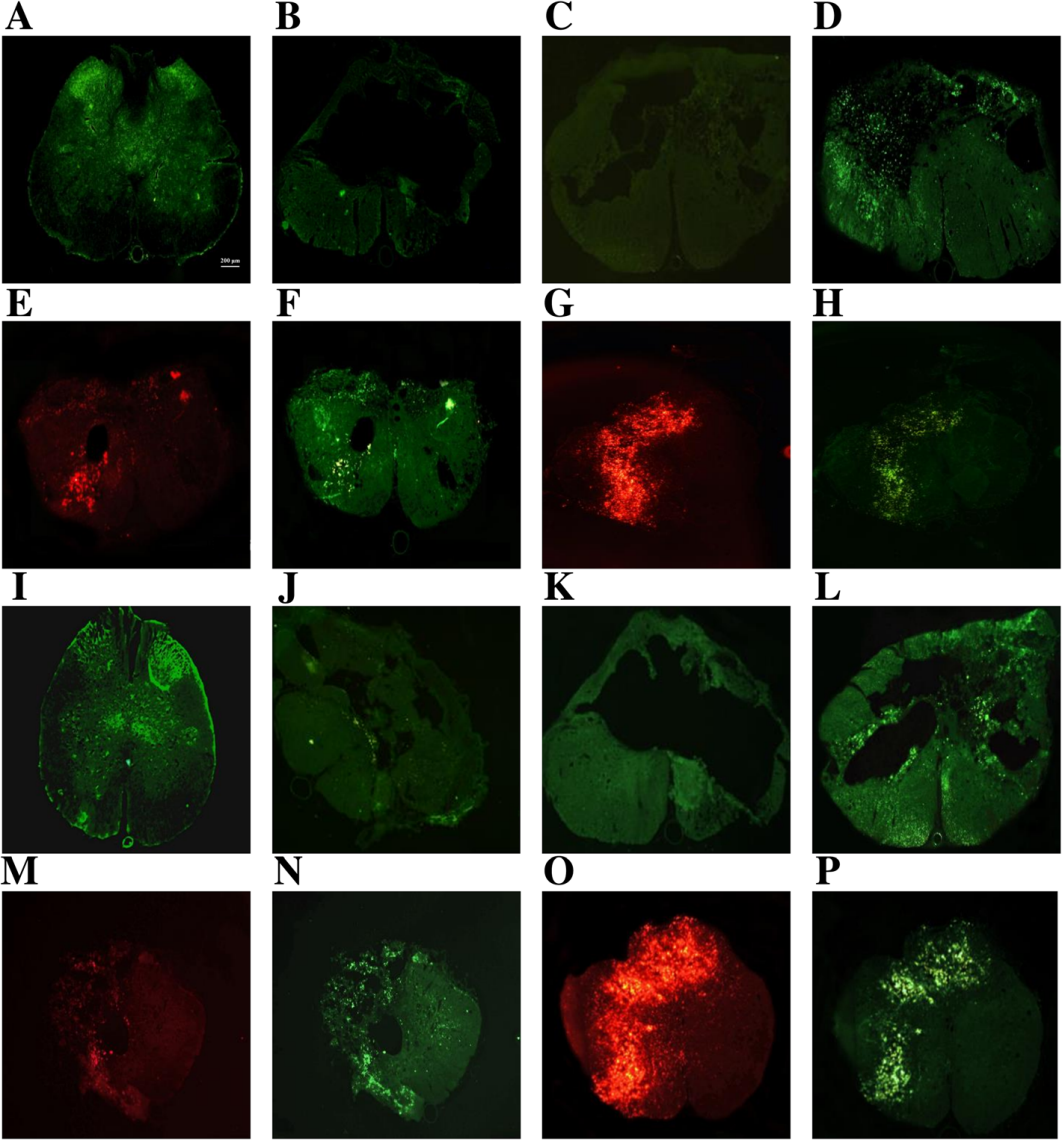
Localization of gamma-aminobutyric acid-B (GABA_B_) type 1 (**a**–**h**) and 2 (**i**–**p**) by immunohistochemistry 4 weeks after spinal cord injury (SCI), spinal cord transverse section (× 10). Control (**a**); SCI (**b**); vehicle (**c**); laser (**d**); hADSCs (cells labeled by DiI, red) (**e**); hADSCs (anti-anti-GABA_B_-1 conjugated whit FITC, green) (**f**); laser+hADSCs (red) (**g**); laser+hADSCs (green) (**h**), Control (**i**); SCI (**j**); vehicle (**k**); laser (**l**); hADSCs (red) (**m**); hADSCs (anti-anti-GABA_B_-2 conjugated whit FITC, green) (**n**); laser+hADSCs (red) (**o**); laser+hADSCs (green) (**p**)

The qualitative assessment showed that SCI result in reduction of GABA receptors (1 and 2) expression in the spinal cord. Treatment by laser and hADSCs increased their expression. The most expression belonged to the laser+hADSC group (Fig. 5).

### Cavity size

**Fig. 6:**
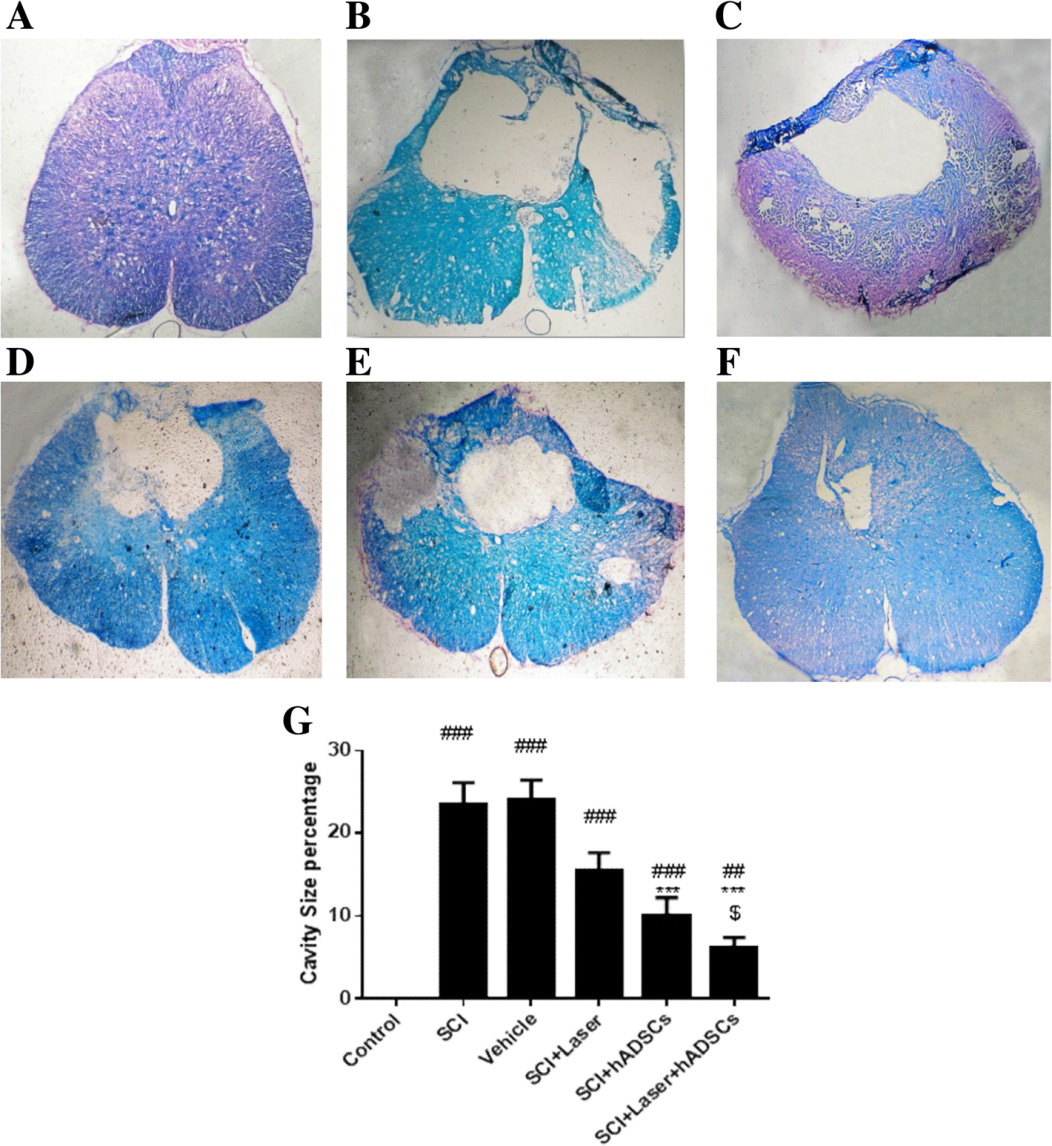
Assessment of cavity size and myelinated area by Luxol Fast Blue (LFB) staining 4 weeks after spinal cord injury (SCI), spinal cord transverse section (× 10). The assessment showed that the largest cavity was observed in the SCI group, while smaller cavities were observed in the combination-treated animals. In addition, the most myelinated area was detected in these animals. Control (**a**); SCI (**b**); vehicle (**c**); laser (**d**); hADSCs (**e**); laser+hADSCs group (**f**); quantitative assay of cavity size (**g**). Data are expressed as mean ± SEM. ^##^*p* < 0.01, ^###^*p* < 0.001, vs control group, ***p < 0.001 vs. SCI group and vehicle; ^$^*p* < 0.05 vs laser

Four weeks after SCI, a large cavity was observed in the injured spinal cord (mean cavity size: 23.4 ± 2.7% for SCI group and mean cavity size: 24.14 ± 2.2% for vehicle groups) (df = 5, 47; F = 24.4; *p* < 0.001). The cavity size of the laser group (mean cavity size: 15.54 ± 2.1%) was not significantly different from that of the SCI group (*p* = 0.09). However, the size of the cavity was significantly smaller in the hADSC group (mean cavity size: 10.16 ± 2.06%; *p* < 0.001) and the laser+hADSC group (mean cavity size: 6.27 ± 1.15%, *p* < 0.001) compared to that in the SCI group. There is a significant difference between the cavity size in the laser and laser+hADSC groups (*p* = 0.02) (Fig. 6).

### Number of axons around the cavity

**Fig. 7:**
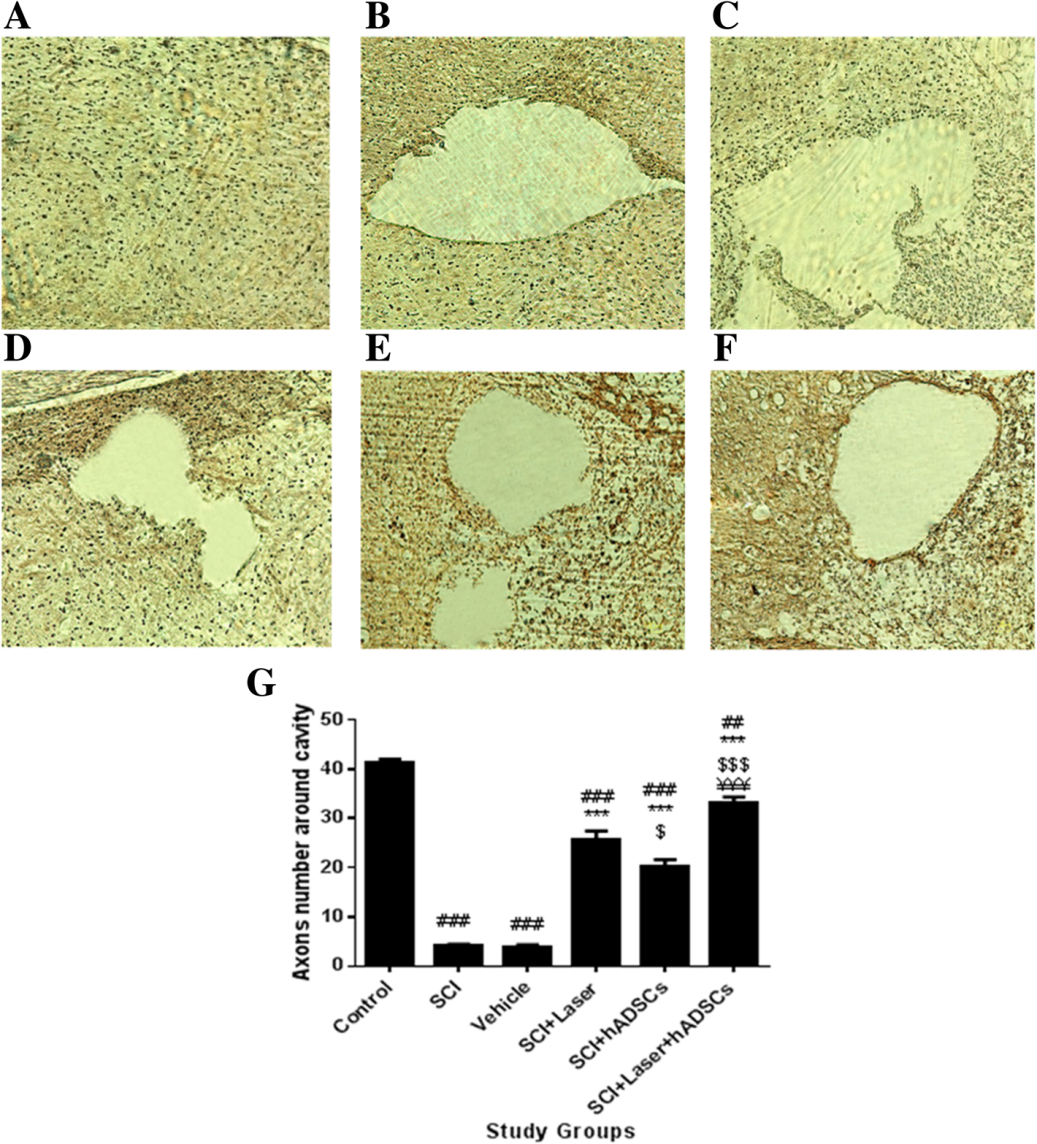
Quantification of axons by Bielschowsky’s silver staining4 weeks after spinal cord injury (SCI), spinal cord transverse section (× 20). Black points show axons. Control (**a**); SCI (**b**); vehicle (**c**), laser (**d**), hADSCs (**e**), laser+hADSCs (**f**) quantitative assay of axons (**g**). SCI led to a reduction in number of axons around damaged areas. hADSCs, laser, and laser+hADSCs were found to have protective effects on the axons and fibers. The laser provides more axon protection than hADSCs. Data are expressed as mean ± SEM. ^###^*p* < 0.001 vs. control group, ***p < 0.01, vs. SCI group and vehicle, ^$^*p* < 0.05, ^$$$^*p* < 0.001 vs. laser group. ^¥¥¥^*p* < 0.001 vs. hADSc group

Bielschowsky’s staining revealed that few axons exist around the cavity in the SCI and vehicle groups (df = 5, 66; F = 154.3, *p* < 0.001) (Fig. 7a–c). Treatment with laser (*p* < 0.0001) and hADSCs (*p* < 0.0001) increased the number of axons around the cavity compared to SCI animals. Co-administration of laser and hADSCs enhanced the number of axons around cavity more than other treatments (*p* < 0.001) (Fig. 7).

## Discussion

The results showed that the combination of laser+hADSCs could significantly improve the motor function recovery and alleviate SCI-induced allodynia and hyperalgesia compared to hADSC-treated animals. Co-administration of laser and hADSCs has significantly increased the BDNF, GDNF, and GABA receptors’ expression. In addition, application of laser+hADSCs has significantly increased the number of axons around the cavity and decreased the cavity size after SCI compared to individual treatments.

GABAergic pathway is the most important mechanism of pain control in the spinal cord [42, 43]. Various studies have shown that increasing the expression of GABA receptors after SCI alleviate neuropathic pain [43, 44]. In the present study, the co-administration of laser+hADSCs, increased the GABA receptor expression. The qualitative assessment showed that the expression of GABA receptors in the laser+hADSC group is higher than that of the individual treatments. hADSCs and laser increase the expression of GABA receptors. It seems that we observed the cumulative effect of hACSCs and laser in increasing the GABA receptors expression.

GSK3β as an apoptotic factor in central nervous system (CNS) inhibits axonal regeneration via increasing the expression of chondroitin sulfate proteoglycan, inducing demyelination and promoting cavity formation [45–48]. On the other hand, it leads to neuropathic pain by changing pro- and anti-inflammatory cytokines [49–51]. In our study, GSK3β expression was increased after SCI, which can be an explanation for increased neuropathic pain. Considering the anti-apoptotic and anti-inflammatory characteristics of hADSCs and laser, it was expected to decrease in GSK3β level in the animals treated with stem cells and laser compared to SCI. However, the expression of GSK3β in hADSC-treated animals (hADSCs and combination groups) was still high. This inconsistency (improvement in neuropathic pain and axonal regeneration despite an increase in GSK3β level) indicates the presence of other mechanisms in this regard.

There is considerable immunodepression after SCI [52]. Therefore, the level of IL-6 in the SCI group did not significantly change compared to control animals. In the animals treated with laser, hADSCs, and combination therapy, the IL-6 level had a significant decrease compared to those treated with SCI, which can be due to the anti-inflammatory characteristic of laser and hADSCs [53, 54]. Studies showed that decreased IL-6 after SCI alleviate the neuropathic pain [55]. In our study, hADSCs and laser caused a significant decrease in the IL-6 level. Therefore, a possible mechanism for anti-nociceptive effect of hADSCs and laser could be related to decrease in IL-6 level. The positive effect of hADSCs and laser on axonal regeneration has been reported in previous studies [22, 56]. It seems that the protective effects of hADSCs and laser on axonal regeneration are not related to the IL-6 pathway, because our treatments decreased IL-6 levels.

The mRNA expression of BDNF in the hADSC and laser+hADSC groups did not have a significant difference with SCI and control groups. Therefore, the neuroregenerative and anti-nociceptive effects of hADSCs appear to have no relationship with BDNF and are probably related to other mechanisms such as the GDNF pathway. However, the level of BDNF in the laser-treated group significantly decreased. In previous studies, it has been proven that increased levels of BDNF are associated with central sensitization and development of neuropathic pain [57]. Therefore, the antonociceptive effect of laser may be due to the decrease of BDNF level.

Transplantation of various cells after SCI has led to increase in GDNF mRNA expression [58–60]. GDNF increases survival of motor and sensory neurons, improves motor function, induces neurogenesis and axonal growth, enhances myelination, and alleviates pain (analgesia effect) [35, 58–62]. In our study also, hADSCs significantly increased GDNF expression. However, laser could not increase GDNF expression compared to SCI and this can be an explanation for observing no decrease in cavity size after laser therapy.

Studies have shown that there is a direct correlation between increase in AQP4 expression and neural death, expansion of edema in the spinal cord, and motor dysfunction following SCI [63–65]. In addition, AQP4 is the confirmation factor for neuroinflammation [66]. There is a shared mechanism between the production of IL-6 and AQP4, and an increase in IL-6 leads to an increase in AQP4 expression and expands inflammation [67]. The studies showed the upregulation of AQP4 could also lead to neuropathic pain [68–71]. In the present study, AQP4 expression is significantly increased in SCI group. Application of laser and hADSCs decreases the expression of AQP4 compared to the SCI group. This finding may be another explanation for protective effect of laser and hADSCs on SCI.

Dorsal root ganglion is intimately associated with neuropathic pain. Therefore, molecular and histological changes in dorsal root ganglion after SCI may provide additional data on exact mechanism of alleviating the effect of laser and hADSCs in SCI. However, the main aim of the present study was to assess the molecular changes in the lesion site. Finally, we suggest the assessing of histological and molecular dorsal root gonglion changes for further studies.

## Conclusion

The results showed that the combination of laser+hADSCs could significantly improve the motor function recovery and alleviate SCI-induced allodynia and hyperalgesia compared to hADSC-treated animals. Therefore, it is suggested that using a combination of laser and hADSCs in future experimental and translational clinical studies could be a promising strategy for alleviating the pain after spinal cord injury.

## Data Availability

The datasets generated and/or analyzed during the current study are not publicly available but are available from the corresponding author on reasonable request.

## Abbreviations

AQP4: Aquaporin 4
BBB: Basso, Beattie, and Bresnahan
*BDNF*: Brain-derived neurotrophic factor
GABA: *Gamma*-aminobutyric acid
GDNF: Glial cell-derived neurotrophic factor
GSK3β: Glycogen synthase kinase 3 beta
hADSCs: Human adipose-derived stem cells
IL-6: Interleukin 6
SCI: Spinal cord injury
